# The regular pattern of metabolite changes in mushroom *Inonotus hispidus* in different growth periods and exploration of their indicator compounds

**DOI:** 10.1038/s41598-022-18631-9

**Published:** 2022-08-23

**Authors:** Zhijun Li, Haiying Bao, Chen Han, Mingjie Song

**Affiliations:** 1 Key Laboratory of Edible Fungi Resources and Utilization, Ministry of Agriculture and Rural Affairs, Jilin Agricultural University, Changchun, Jilin, 130118 China; 2 College of Chinese Medicinal Materials, Jilin Agricultural University, Changchun, 130118 China

**Keywords:** Metabolomics, Natural products

## Abstract

*Inonotus hispidus* is a valuable and rare edible and medicinal mushroom with extremely high nutritional and medicinal value. However, there is no holistic insight to elucidate the molecular basis of the differentiated usage and accurate annotation of physiological maturity to fluctuating yields and quality. This study aimed to figure out the fruiting bodies' metabolites change regulation and potential maturating indicators to distinguish different quality *I. hispidus*. We applied non-targeted ultra-high performance liquid chromatography and high-resolution mass spectrometry combined and with multivariate analysis and analyzed cultivated and wild mushroom *I. hispidus* in different growth periods (budding, mature and aging). With the fruiting bodies maturating, 1358 metabolites were annotated, 822 and 833 metabolites abundances changed greater than or equal to 1 time from the budding period to the aging period in abundance in cultivated and wild, the total polysaccharides, crude fat, total flavonoids, and total terpenes increased at first and then decreased. Total amino acids, crude protein, and total polyphenols decreased, while the total steroids increased linearly. The change of metabolites showed certain regularity. Metabolic pathways enrichment analysis showed that these metabolites are involved in glycolysis, biosynthesis of amino acids, organic acid metabolism, glycine-serine-and-threonine metabolism, tricarboxylic acid cycle, purine metabolism, and pyrimidine metabolism. In addition, ergosterol peroxide and (22E)-ergosta-4,6,8(14),22-tetraen-3-one can be used as indicator compounds, and their contents increase linearly with the fruiting bodies of *I. hispidus’* physiological maturation. This comprehensive analysis will help to evaluate the edible values and facilitate exploitation in mushroom *I. hispidus.*

## Introduction

The mushroom *Inonotus hispidus* (Bull.: Fr.) P. Karst. (*I. hispidus*) is an edible and medicinal mushroom, and the fruiting body is not only used as a food material but commonly soaked in water as a health drink in China^1^. It is a traditional Chinese medicine "*Sanghuang*" that was described in ancient Chinese Materia medica books in China, such as Shennong's Classic of Materia Medica and Compendium of Materia Medica. It is used in folk culture as an astringent, a diuretic, and for the treatment of mouth ulcers and inflammation^2^. Novel scientific investigations revealed anti-tumor^3,4^, improving human immunity^5^, anti-inflammatory^6^, antioxidant^7^, antiviral^8^, and hypolipidemic activity^9^, and it has great potential application value and development potential in the field of functional food and medicine. The appearance of the fruiting bodies of *I. hispidus* is golden in the budding period, which can be used as delicious food, but the yield is low. The fruiting bodies in the mature period appears yellowish-brown, while the fruiting body in the aging period contains a large amount of melanin^10^, which is grayish-black, and the yield is relatively high, but the harvest time is longer than the budding period. As to the fruiting bodies of *I. hispidus* in different growth periods, the pharmacological effects are also different.

At present, in the research and commercial production of functional food related to *I. hispidus*, there is a lack of research on the variation of metabolite abundance in different growth periods of *I. hispidus*. Because of the lack of measurable indicators that can characterize the physiological maturity of *I. hispidus*, which leads to the subjective evaluation of the physiological maturity and the fluctuation of the yield and quality of *I. hispidus* and the difference in production efficiency of different producers. The quality of *I. hispidus* can be understood in this way, the fruiting bodies with various metabolic components that can meet the industrial needs of pharmaceutical production are considered to be high-quality *I. hispidus* samples. For example, in industrial production, the main purpose is to obtain the total amino acid components in the fruiting bodies of *I. hispidus*, then the fruiting body with a relatively high content of total amino acids is considered to be of high quality. The high quality of *I. hispidus* fruiting bodies can’t be effectively selected in functional food, medical research and production practice, which has become one of the important factors restricting industrial production. Therefore, there is an urgent need for an indicator of fruiting bodies' maturation, to provide a basis for production and research.

Metabolomics is a new discipline that has developed rapidly following genomics, transcriptomics, and proteomics^11–13^. It aims to detect the overall trajectory of endogenous metabolites in organisms or cells under specific conditions to reflect the pathological and physiological processes of organisms, and some differential metabolites detected have become potential markers that characterize the pathological and physiological states of organisms^14^. Metabolomics has been widely used in many fields, such as animal and plant metabolism^15,16^, microbial metabolism^17,18^, disease diagnosis^19–21^, and drug development^22^. In recent years, metabolomics technology has been gradually applied to the field of edible and medicinal mushrooms to study metabolic profiling^23–25^. Those studies were carried out scientifically by the method of metabolomics, such as analyzing the metabolic profiling changes in mycelia collected at *Pleurotus tuoliensis* day 0, and day 35 under different temperatures (17 and 29 °C) by using GC–MS-based metabolomics^26^, and targeted metabolic profiling annotate changes in the *lenticular edodes* mycelial metabolome under high-temperature stress^27^.

So far, the metabolic profiling of different periods of *I. hispidus* mushroom has largely remained unexplored, and the grading has been confused by producers, traders, consumers, researchers, and related regulatory bodies. Almost all of them in the market were objective for discrimination of growth period based on phenotypic information and physical properties such as individual weight, tissue size, color, water content, etc. We hypothesized that the different qualities of *I. hispidus* differ in their metabolomic profiles, and they could be distinguished by their chemical constituents.

This study aimed to figure out the fruiting bodies' metabolites change regulation and potential maturating indicators to distinguish different quality *I. hispidus*. Through the potential indicator compounds of physiological maturity of it as a reference, to determine the best harvest time and guide the production practice, to maximize the yield and quality. We found that two metabolites were closely related to their physiological maturity, indicating that they have the potential to be used as markers to distinguish different quality grades. The result was helpful to evaluate *I. hispidus* fruiting body quality and facilitate exploitation in its consumption and processing.

## Results

### Principal component analysis of cultivated and wild *Inonotus hispidus* fruiting bodies in different growth periods

**Figure 1:**
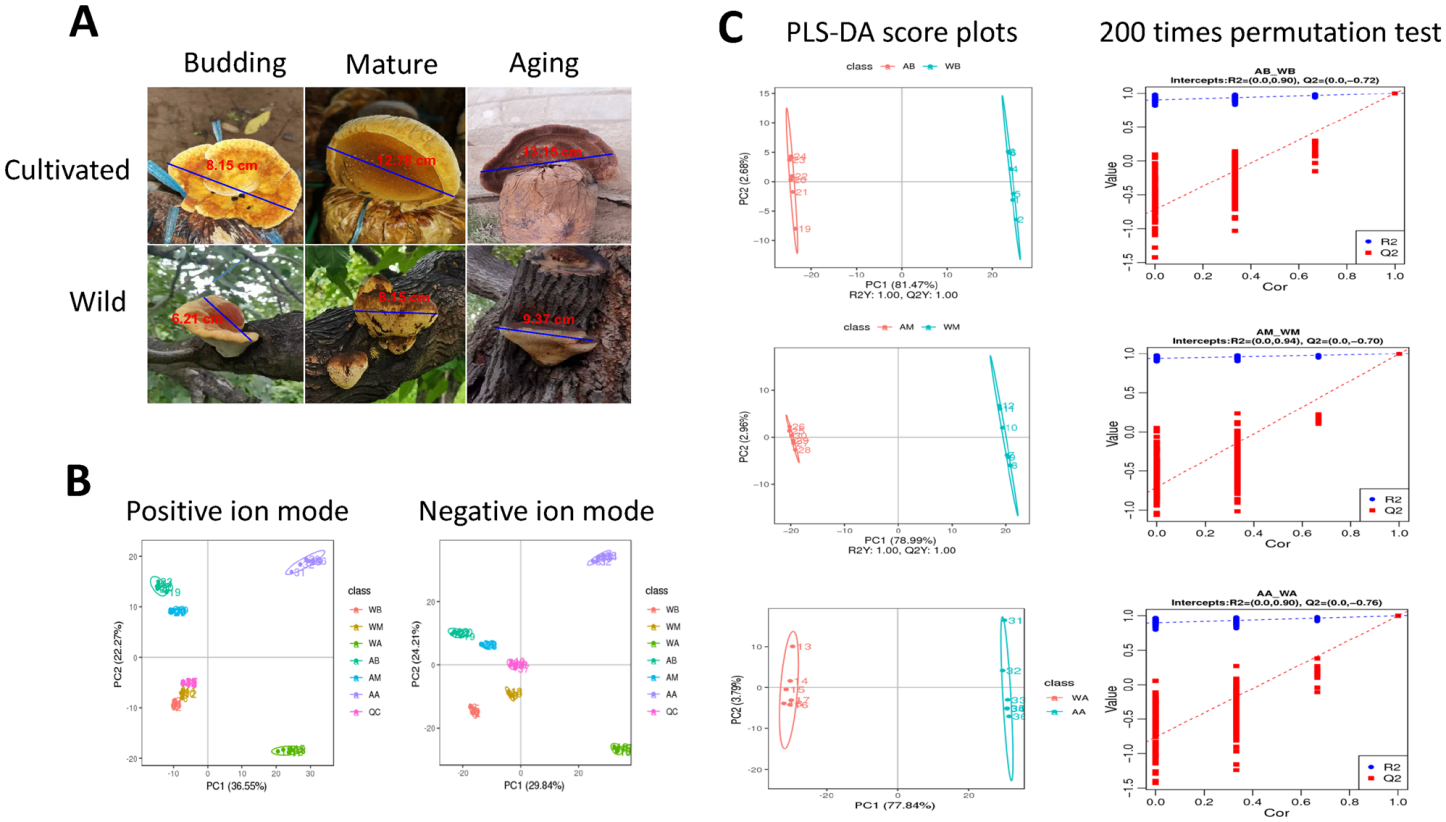
The *Inonotus hispidus* samples and principal component analysis score chart. (**A**) The fruiting bodies of *Inonotus hispidus* in different growth periods. (**B**) Principal component analysis score chart. (**C**) Partial Least-Squares Discriminant Analysis (PLS-DA) score plots from metabolite profiles for fruiting bodies and a 200 times permutation test of PLS-DA models for it. Score plots from metabolite profiles for fruiting bodies and a 200 times permutation test of PLS-DA models for it. Note: AB, AM, and AA are samples of fruiting bodies of cultivated *Inonotus hispidus* in budding, mature and aging growth periods, respectively. WB, WM, and WA are samples of fruiting bodies of wild *Inonotus hispidus* in budding, mature and aging growth periods, respectively.

First, unsupervised Principal Component Analysis (PCA) was applied to assess the overall distribution among all samples and the stability of the entire analysis process. As shown in (Fig. 1B), all the quality control samples are clustered together, showing good analytical stability and experimental reproducibility. In our principal component analysis model, 6 groups of samples are well separated. Among them, the samples of wild *I. hispidus* in the budding period (WB), wild *I. hispidus* in the mature period (WM), artificial cultivated *I. hispidus* in the budding period (AB), and cultivated *I. hispidus* in the mature period (AM) are clustered together closely, while the samples of wild *I. hispidus* in aging period (WA) and cultivated *I. hispidus* in aging period (AA) deviate slightly. These results are consistent with our previous conclusion that there are great differences in the types of metabolites in different growth periods of the fruiting bodies of *I. hispidus*, and there are great differences in the abundance of metabolites between the fruiting bodies in the aging period and the other two periods. The wild and cultivated *I. hispidus* mushroom in different growth periods as shown in Fig. 1A.

### Partial least-squares discriminant analysis

To determine the budding, mature, and aging fruiting bodies of cultivated and wild *I. hispidus*, and the metabolic differences among 6 groups of samples, the supervised partial least square discriminant analysis model was used to further optimize the population separation of fruiting bodies. The comparison between paired AB, AM, and AA samples of Partial Least-Squares Discriminant Analysis (PLS-DA) fruiting bodies showed that there were significant differences in metabolism among different categories in each pairwise comparison of the first component (Fig. 1C). The partial least squares model has high R2Y and Q2 values, which indicated an excellent fit and satisfactory predictive power. A permutation test was used to evaluate the possible overfitting of the PLS-DA model. In this study, a 200-time permutation test was performed. R2 intercept of fruiting body AB and WB samples is 0.90 and R2 intercept of WM samples is 0.94. R2 intercept of AA and WA samples is 0.90, while Q2 intercept is − 0.72, − 0.70, and − 0.76, respectively. Figure 1C indicates that the PLS-DA model showed no overfitting and was credible. The annotation of differential metabolites between pairs of samples was performed using VIP values and confirmed by the non-parametric Mann–Whitney U test.

### Comparative analysis of differential metabolites

**Figure 2:**
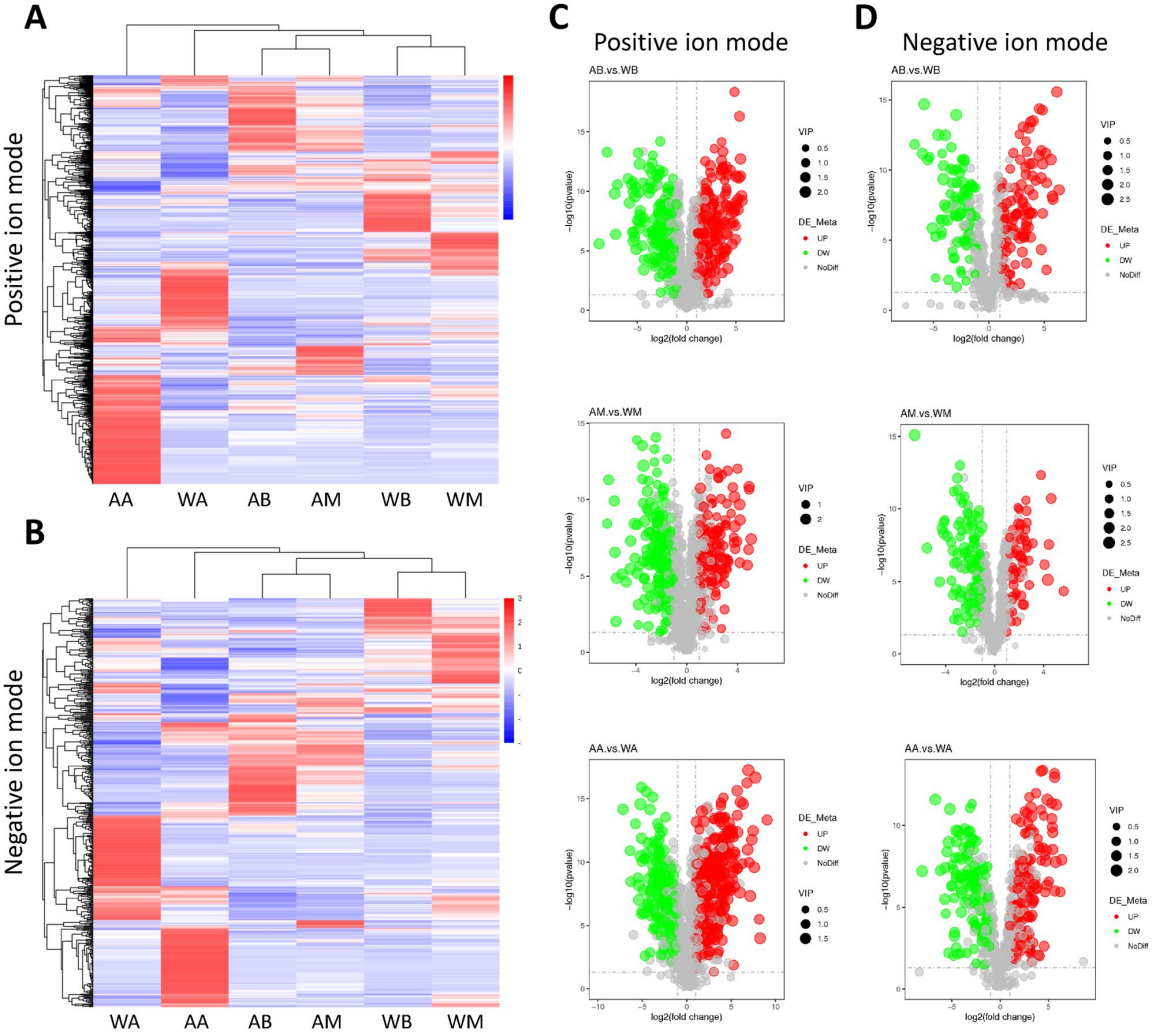
Metabolite profiling of different growth periods of fruiting bodies of cultivated and wild *Inonotus hispidus* are compared at budding, mature and aging stages (the data used in heatmaps are Z-score of expressions). Note: AB, AM, and AA are samples of fruiting bodies of cultivated *Inonotus hispidus* in budding, mature and aging growth periods, respectively. WB, WM, and WA are samples of fruiting bodies of wild *Inonotus hispidus* in budding, mature and aging growth periods, respectively. Heat map of cultivated *Inonotus hispidus* in positive ion mode (**A**), negative ion mode (**B**). Each fruiting bodies sample is visualized in a single column and each metabolite is represented by a single row. blue colors indicate lower metabolite concentration, while red colors show enhanced metabolite levels. Volcano plot in positive ion mode (**C**), negative ion mode (**D**). Each point in the Volcano plot represents a metabolite, with significantly up-regulated metabolites represented by red dots and significantly down-regulated metabolites represented by green dots.

**Figure 3:**
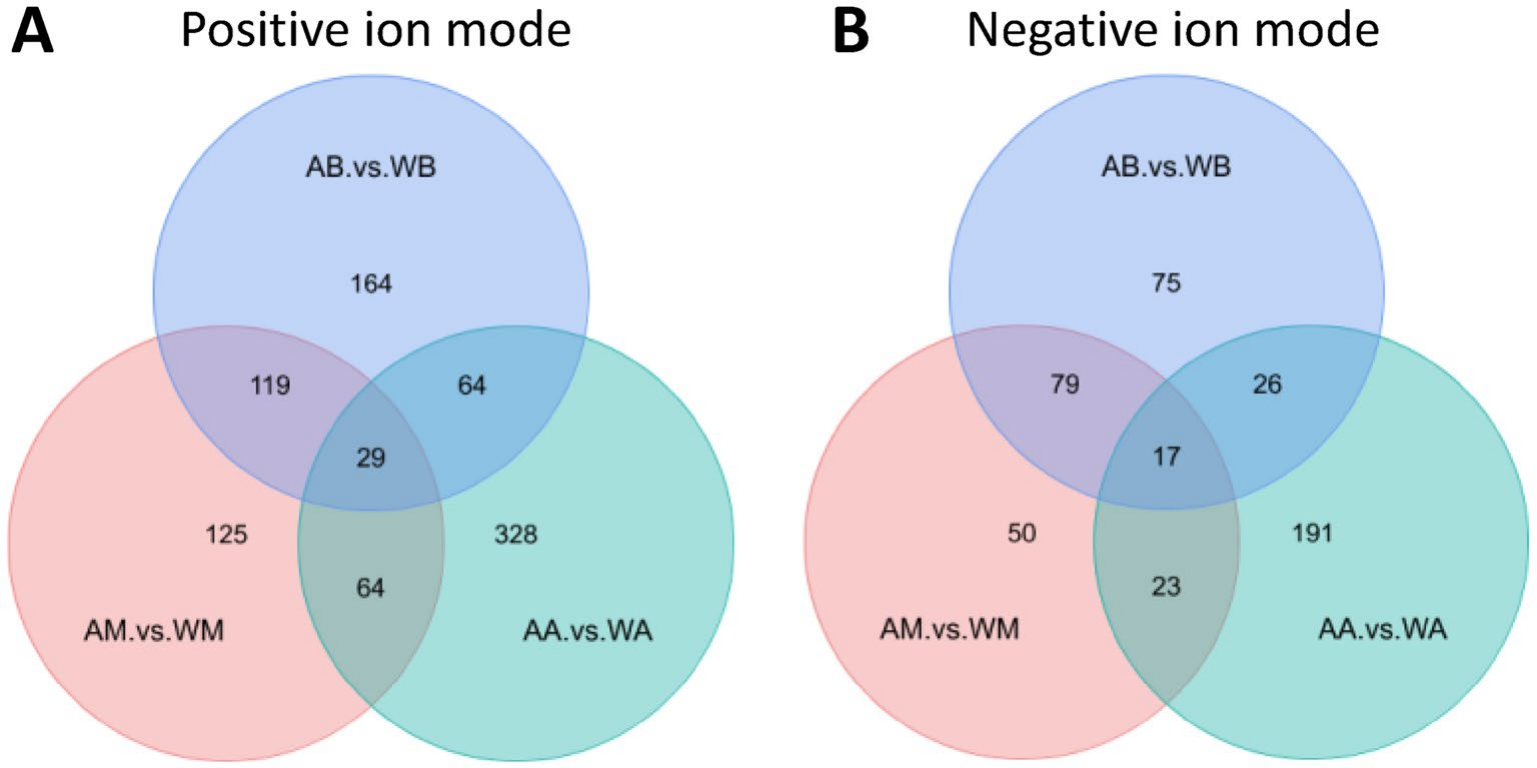
Venn diagram analysis of differential metabolites of cultivated and wild *Inonotus hispidus* in different growth periods comparative combinations. (**A**) Under the positive ion mode and (**B**) under the negative ion mode. Note: AB, AM, and AA are samples of fruiting bodies of cultivated *Inonotus hispidus* in budding, mature and aging growth periods, respectively. WB, WM, and WA are samples of fruiting bodies of wild *Inonotus hispidus* in budding, mature and aging growth periods, respectively.

With the fruiting bodies maturating, 1358 metabolites were annotated, 822 and 833 metabolites' abundances changed greater than or equal to 1 time from the budding period to the aging period in abundance in cultivated and wild (as shown in Supplementary Materials S1). Taking the expression of all metabolites annotated as data, the samples were compared by single-factor analysis of variance (one-way ANOVA). Corrected by the Benjamini–Hochberg method, the metabolites were screened for differential expression with P-value 0.05 as the threshold, and the metabolites were classified according to the expression between samples. The heat-map of different metabolites of cultivated and wild mushroom *I. hispidus* can be seen in (Fig. 2A,B). Vertical clustering is the clustering of samples, and horizontal clustering is the clustering of metabolites. The shorter the clustering branches are, the higher the similarity is. Through horizontal comparison, we can see the relationship of metabolite abundance clustering between groups. The results of the heat map showed that the abundance of metabolites varied greatly from the budding period to the mature period and then to the aging period, no matter whether in cultivated or wild ones. Because the abundance of metabolites is different in different growth periods, the pharmacological activity and value of *I. hispidus* are different, which shows that it is necessary to use scientific methods to accurately judge the best harvest time. In addition, the volcano plot of differential metabolites showed that there were great differences in metabolites between cultivated and wild mushroom *I. hispidus* in the same period. Volcano plot indicating upregulated and downregulated (Fig. 2C,D). In different growth periods (budding, mature and aging), the Venn diagram of the differential metabolites in the fruiting bodies of cultivated and wild *I. hispidus* showed the overlapping and differential parts of the metabolites. In this study, the Venn diagram analysis of the differential metabolites of the three comparative combinations is shown in Fig. 3 (Venn diagram in positive ion mode as shown in Supplementary Materials S2, and in negative ion mode as shown in Supplementary Materials S3).

### Sugars, amino acids metabolites, and oligopeptides were annotated in *Inonotus hispidus*

In this study, 20 sugars,12 amino acids with different configurations (including 4 essential amino acids and 8 non-essential amino acids), and 24 oligopeptides were annotated in mushroom *I. hispidus*. With the physiological maturity of the fruiting body, most of the sugars and amino acid content of cultivated *I. hispidus* decreased. The abundance of arginine increased at first and then decreased. The abundance of proline decreased at first and then increased. However, with the physiological maturity of the fruiting body, the abundance of phenylalanine, proline, aspartic acid, and tyrosine increased at first and then decreased, and the abundance of other amino acids decreased in the wild *I. hispidus*. These changes are worth thinking about and studying, and the collection of these substances is not only the characteristic components of *I. hispidus*, but also the material basis to play a unique biological function. The detailed changes of amino acids and oligopeptides are shown in Table 1.

**Table 1:** Summary of sugars, amino acids metabolites, and oligopeptides were annotated in *Inonotus hispidus.*

Compound class	NO	Metabolites	Fold change					
			WM/WB	WA/WB	WA/WM	AM/AB	AA/AB	AA/AM
Sugars	1	Fructose	0.47	0.23	0.48	1.39	2.07	1.49
	2	Lyxose	2.63	2.15	0.82	0.96	1.05	1.10
	3	Tagatose	0.79	0.52	0.67	1.07	1.74	1.63
	4	Arabitol	5.79	1.62	0.28	0.98	0.06	0.07
	5	Deoxyribose 5-Phosphate	1.46	0.64	0.44	0.58	1.65	2.84
	6	Fructose 1,6-bisphosphate	1.39	0.44	0.31	0.44	0.35	0.79
	7	Fructose 6-phosphate	1.52	5.19	3.41	1.39	9.25	6.68
	8	Glucosamine	0.65	0.04	0.06	0.88	1.01	1.15
	9	1-O-(3,4,5-Trimethoxybenzoyl)-beta-L-galactopyranose	0.39	0.25	0.65	1.18	1.11	0.94
	10	Cyclic ADP-ribose	1.28	0.50	0.39	0.31	0.08	0.26
	11	Mannitol	0.33	0.16	0.49	0.43	0.21	0.50
	12	Threose	1.10	0.42	0.38	0.98	0.54	0.55
	13	Arabinose	0.84	0.73	0.87	0.86	0.83	0.96
	14	Fucose	1.73	0.97	0.56	2.10	1.01	0.48
	15	Iditol	1.88	0.54	0.28	2.25	0.29	0.13
	16	Maltotriose	1.34	3.49	2.60	2.58	0.80	0.31
	17	Trehalose-6-phosphate	1.01	1.44	1.42	0.38	0.09	0.23
	18	Xylitol	0.18	0.14	0.76	0.88	0.86	0.97
	19	Trehalose	0.74	0.47	0.63	0.02	0.04	1.48
	20	Lactose	1.20	0.69	0.58	0.92	0.14	0.16
Essential amino acid	21	Lysine	0.23	0.01	0.04	0.30	0.05	0.16
	22	Phenylalanine	3.96	0.87	0.22	0.40	0.88	2.23
	23	Threonine	0.65	0.35	0.54	0.90	0.46	0.52
	24	Methionine	0.87	0.72	0.83	2.12	0.24	0.12
Non-essential amino acids	25	Asparagine	0.47	0.04	0.08	0.57	0.07	0.12
	26	Arginine	0.44	0.01	0.03	1.05	0.11	0.11
	27	Serine	0.52	0.03	0.05	0.86	0.14	0.16
	28	Proline	2.12	0.28	0.13	0.81	1.29	1.59
	29	Aspartic acid	1.16	0.69	0.59	0.52	0.24	0.45
	30	Glutamic acid	0.69	0.07	0.10	0.60	0.39	0.65
	31	Histidine	0.41	0.03	0.06	0.46	0.42	0.91
	32	Tyrosine	3.95	1.17	0.30	0.79	0.36	0.45
Oligopeptides	33	Anserine	1.18	0.65	0.55	1.01	1.24	1.23
	34	Ala-Gln	0.69	0.23	0.33	1.35	3.69	2.73
	35	Ala-Ile	1.05	0.87	0.83	1.00	1.17	1.18
	36	Ala-Leu	1.39	0.85	0.61	0.99	0.53	0.53
	37	Ala-trp	1.05	0.12	0.12	2.89	4.77	1.65
	38	Ala-Val	1.21	0.36	0.29	0.57	0.64	1.12
	39	Asp-Phe	0.63	0.10	0.17	0.72	0.87	1.22
	40	Ala- Ala	2.84	0.90	0.32	0.56	1.16	2.06
	41	Gamma-Glu-Glu	1.41	0.88	0.62	0.81	2.98	3.67
	42	Gamma-Glu-Leu	1.02	0.42	0.42	1.02	1.10	1.07
	43	Glu-Gln	3.78	0.12	0.03	0.63	2.09	3.32
	44	Glu-Thr	0.99	0.15	0.15	1.01	0.78	0.77
	45	Glu-Val-Phe	1.33	1.37	1.03	2.10	2.39	1.14
	46	Gly-Phe	1.59	0.43	0.27	1.00	1.96	1.96
	47	Gly-Tyr	1.37	0.48	0.35	2.03	1.08	0.53
	48	Gly-Tyr-Ala	2.23	0.14	0.06	2.19	2.34	1.07
	49	H-Gly-Pro-OH	1.74	0.22	0.13	1.43	1.18	0.83
	50	N-Acetyl-Asp-Glu	1.45	0.16	0.11	1.06	0.38	0.36
	51	Phe-Phe	1.39	0.92	0.66	0.94	0.09	0.09
	52	Phe-Pro	1.09	0.12	0.11	1.43	0.61	0.43
	53	Pro-Leu	1.41	0.10	0.07	0.86	0.52	0.60
	54	Thr-Leu	0.46	0.04	0.08	0.62	1.16	1.86
	55	Tyr-Tyr	0.85	0.09	0.10	0.74	0.09	0.13
	56	Val-Ser	0.41	0.02	0.04	0.67	1.29	1.92

WM/WB: The ratio of the mature period to budding period of wild *Inonotus hispidus*. WA/WB: The ratio of the mature period to budding period of wild *Inonotus hispidus*. WA/WM: The ratio of the aging period to mature period of wild *Inonotus hispidus.*

AM/AB: The ratio of the mature period to budding period of artificially cultivated *Inonotus hispidus*. AA/AB: The ratio of the aging period to budding period of artificially cultivated *Inonotus hispidus*. AA/AM: The ratio of the aging period to mature period of artificially cultivated *Inonotus hispidus.*

### Sterols, phenols, flavonoids, and terpenoids metabolites were annotated in *Inonotus hispidus*

With the physiological maturity of the *I. hispidus* fruiting body, most steroids and phenols metabolites increased over the whole period. Most flavonoid metabolites increased at first and then decreased. Among sterols and their derivatives metabolites, the abundance of ergosta-5,7,9(11),22-tetraen-3-beta-ol increased at first and then decreased in cultivated *I. hispidus.* But in the wild *I. hispidus* increased with the fruiting body physiological maturity. However, the abundance of ergosterol peroxide of cultivated *I. hispidus* decreased in the whole period. The abundance of ouabain of wild *I. hispidus* increased at first and then decreased, but in wild samples, it decreased first and then increased. These changes will directly affect the quality of *I. hispidus* in different growth periods. Detailed changes about sterols, phenols, flavonoids, and terpenoids metabolites in mushroom *I. hispidus* are shown in Table 2.

**Table 2:** Summary of sterols, phenols, flavonoids, and terpenoids metabolites were annotated in *Inonotus hispidus.*

Compound class	NO	Metabolites	Fold change					
			WM/WB	WA/WB	WA/WM	AM/AB	AA/AB	AA/AM
Sterols and their derivatives	1	2,3,14,20,22-Pentahydroxyergost-7-en-6-one	0.59	0.41	0.69	0.88	3.60	4.09
	2	Ergosta-5,7,9(11),22-Tetraen-3-beta-ol	2.26	0.20	0.09	1.62	2.93	1.81
	3	Ouabain	1.65	0.50	0.30	0.90	2.06	2.28
	4	Strophanthidin	1.13	1.33	1.18	0.81	14.61	18.02
	5	Ergosterol peroxide	2.08	4.15	1.99	2.08	5.68	2.73
Phenols and their derivatives	6	2-Methoxyresorcinol	1.88	2.41	1.28	1.50	1.56	1.04
	7	2-Naphthol	1.39	0.96	0.69	1.02	0.01	0.01
	8	2-Phenylphenol	1.14	0.20	0.18	1.03	0.12	0.12
	9	3-[2-(3-Hydroxyphenyl)ethyl]-5-methoxyphenol	2.92	5.28	1.80	0.79	1.59	2.02
	10	3-Methoxytyramine	1.26	1.37	1.08	0.92	4.62	5.04
	11	4-Butylresorcinol	1.24	1.00	0.81	1.47	0.56	0.38
	12	4-Methylphenol	1.39	0.94	0.68	0.95	0.91	0.96
	13	Alternariol	1.63	3.33	2.05	0.73	0.13	0.17
	14	8-THC-d9	0.74	0.45	0.61	1.19	1.05	0.89
	15	9-THC-d3	0.66	21.29	32.27	1.84	14.54	7.92
	16	Dithranol	3.14	3.79	1.21	1.37	1.37	1.00
	17	Flavin mononucleotide (FMN)	1.32	0.91	0.69	1.08	0.10	0.09
	18	Tocopherol	1.01	1.21	1.20	1.00	1.24	1.24
	19	Hematoxylin	1.62	0.74	0.46	1.37	0.06	0.04
	20	Homovanillic acid	2.61	1.46	0.56	0.42	0.87	2.08
	21	Isorhapontigenin	1.10	30.09	27.30	0.96	1.05	1.09
	22	Nor-9-carboxy-δ9-THC	1.28	2.10	1.64	1.62	4.93	3.04
	23	O-Cresol	1.24	0.99	0.80	1.09	1.86	1.71
	24	O-Desmethylnaproxen	0.98	1.89	1.93	1.29	1.34	1.04
	25	Paracetamol	0.63	0.18	0.28	0.89	0.30	0.34
	26	Phloroglucinol	0.80	0.20	0.25	1.11	0.12	0.11
	27	Pyrogallol	0.01	1.48	217.96	0.79	0.80	1.01
	28	THC	1.36	0.16	0.12	1.40	0.41	0.29
Flavones	29	2-(2,4-dihydroxyphenyl)-3,5,7-trihydroxy-4H-chromen-4-one	1.94	0.71	0.37	3.01	0.85	0.28
	30	3,5,7-trihydroxy-2-phenyl-4H-chromen-4-one	1.22	1.13	0.92	1.62	0.59	0.37
	31	Apigenin	4.92	3.33	0.68	1.20	0.61	0.51
	32	Galangin	0.83	0.26	0.32	1.23	0.13	0.10
	33	Isorhamnetin	1.85	1.66	0.90	1.95	0.06	0.03
	34	Kaempferol	0.91	1.39	1.53	0.46	0.16	0.36
	35	Luteolin	1.03	0.94	0.91	0.28	0.14	0.51
	36	Myricetin	1.24	6.37	5.15	1.21	0.63	0.52
	37	Puerarin	1.03	1.20	1.16	23.78	0.23	0.01
	38	Purpurin	4.12	4.27	1.04	1.68	0.15	0.09
	39	Quercetin	1.41	0.53	0.37	2.29	0.03	0.02
	40	Wogonin	0.91	0.81	0.88	0.78	0.17	0.22
Flavonoids	41	3',5,7-Trihydroxy-4'-methoxyflavanone	0.15	0.41	2.79	0.79	0.12	0.15
	42	4',7-Dihydroxyflavanone	2.62	0.05	0.02	0.79	0.83	1.06
	43	7-hydroxy-3-(4-methoxyphenyl)-4H-chromen-4-one	41.37	6.24	0.15	1.47	1.76	1.20
	44	Biochanin A	0.70	0.42	0.60	0.68	0.89	1.31
	45	Catechin	0.89	0.14	0.16	0.64	0.26	0.41
	46	Equol	0.50	0.05	0.10	1.18	0.09	0.07
	47	Eriodictyol	1.50	1.85	1.23	0.97	0.04	0.04
	48	Hesperetin	2.30	1.11	0.48	1.94	0.67	0.34
	49	Pinocembrin	1.29	3.27	2.54	1.17	2.80	2.38
	50	Sakuranetin	1.24	1.17	0.95	0.87	2.85	3.27
Isoflavonoids	51	Coumestrol	0.93	0.29	0.31	0.86	0.08	0.09
	52	Daidzein	36.29	3.80	0.10	1.47	0.86	0.59
	53	Glycitein	1.62	1.52	0.94	1.39	0.48	0.35
	54	Rotenone	1.35	0.34	0.25	1.01	0.05	0.05
Terpenoids and their derivatives	55	Turmerone	2.71	156.80	57.92	2.17	2.06	0.95
	56	2,6-Di-tert-butyl-1,4-benzoquinone	0.91	9.83	10.84	2.07	13.30	6.42
	57	Farnesene	1.80	2.53	1.40	1.26	2.22	1.77
	58	Betulin	0.74	0.39	0.52	0.03	0.03	1.05
	59	Carvone	1.29	1.89	1.46	1.14	1.87	1.64
	60	Camphor	6.01	0.97	0.16	0.94	1.56	1.66
	61	Limonin	1.17	0.74	0.63	1.11	0.36	0.33
	62	Linalool	0.85	5.84	6.88	1.27	4.51	3.54
	63	Perillartine	2.02	0.57	0.28	1.42	1.83	1.29
	64	P-Mentha-1,3,8-triene	2.17	1.38	0.63	0.56	0.74	1.32
	65	Ursolic acid	2.36	3.94	1.67	2.08	3.25	1.57

WM/WB: The ratio of the mature period to budding period of wild *Inonotus hispidus*. WA/WB: The ratio of the mature period to budding period of wild *Inonotus hispidus*. WA/WM: The ratio of the aging period to mature period of wild *Inonotus hispidus.*

AM/AB: The ratio of the mature period to budding period of artificially cultivated *Inonotus hispidus*. AA/AB: The ratio of the aging period to budding period of artificially cultivated *Inonotus hispidus*. AA/AM: The ratio of the aging period to mature period of artificially cultivated *Inonotus hispidus.*

### Changes of total polysaccharides, total amino acids, crude protein, crude fat, total sterols, total polyphenols, total flavonoids, and total terpenes in fruiting bodies of *Inonotus hispidus* in different growth periods

**Figure 4:**
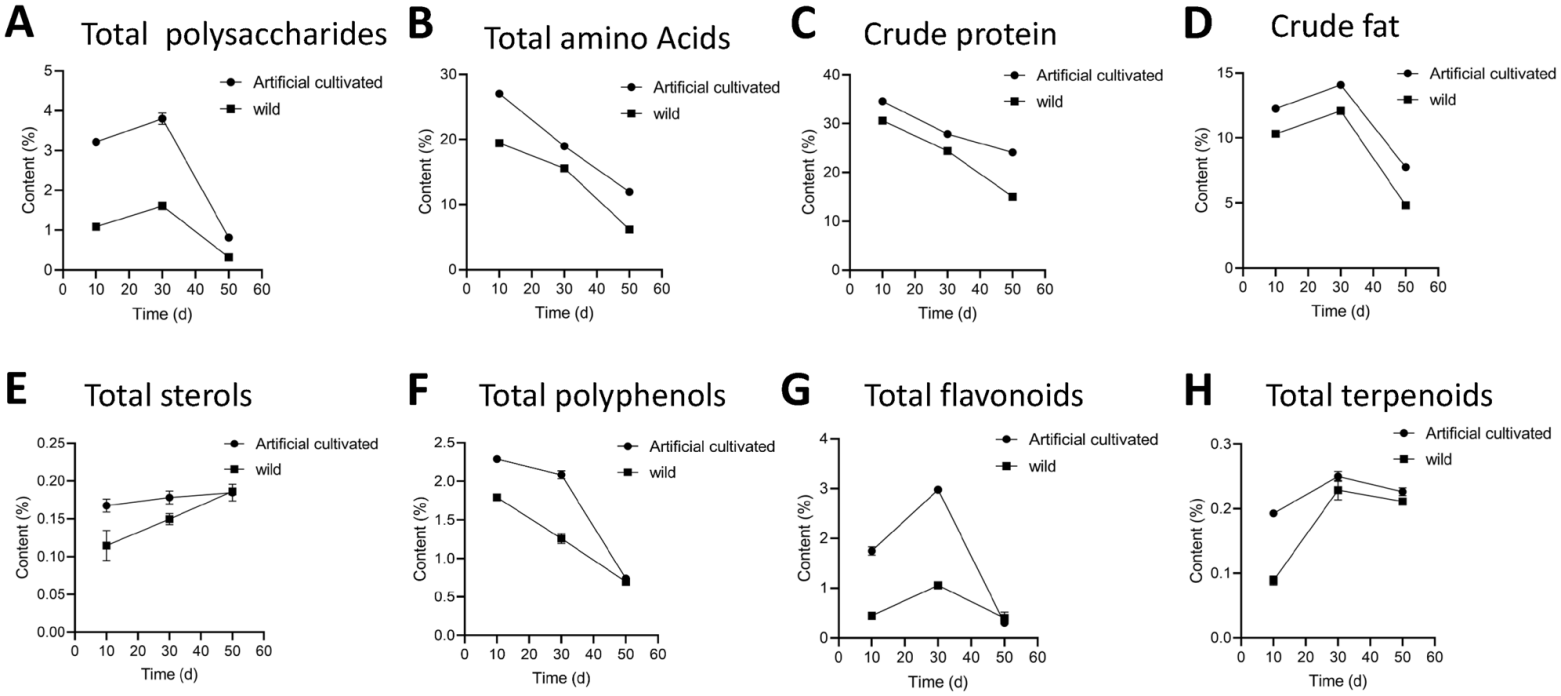
Line diagram of content changes of total polysaccharides, total amino acids, total proteins, crude fats, total steroids, total polyphenols, total flavonoids, and total terpenoids in *Inonotus hispidus.*

With the physiological maturity of the *I. hispidus* fruiting body, the metabolites showed a regular change trend, and this changing trend occurred not only in cultivated *I. hispidus*, but also in wild. The results showed that the fruiting body of *I. hispidus* is in the process from the budding period to the mature period and then to the aging period, the abundance of total polysaccharides, crude fat, total flavonoids, and total terpenes increased at first and then decreased. Interestingly, total amino acids, crude protein, and total polyphenols decreased during the growth periods, while the total steroids increased linearly. The analysis of the content and change regular of the metabolites showed distinct changes in abundance in cultivated and wild. From an overall point of view, the changes of metabolites in cultivated were more stable and the content of metabolites higher than the wild ones. Therefore, we speculate that the cultivated *I. hispidus* may be more suitable for the function food or medicines. The 8 kinds of primary and secondary metabolites changed trend were shown in Fig. 4.

### Metabolic pathway analysis

**Figure 5:**
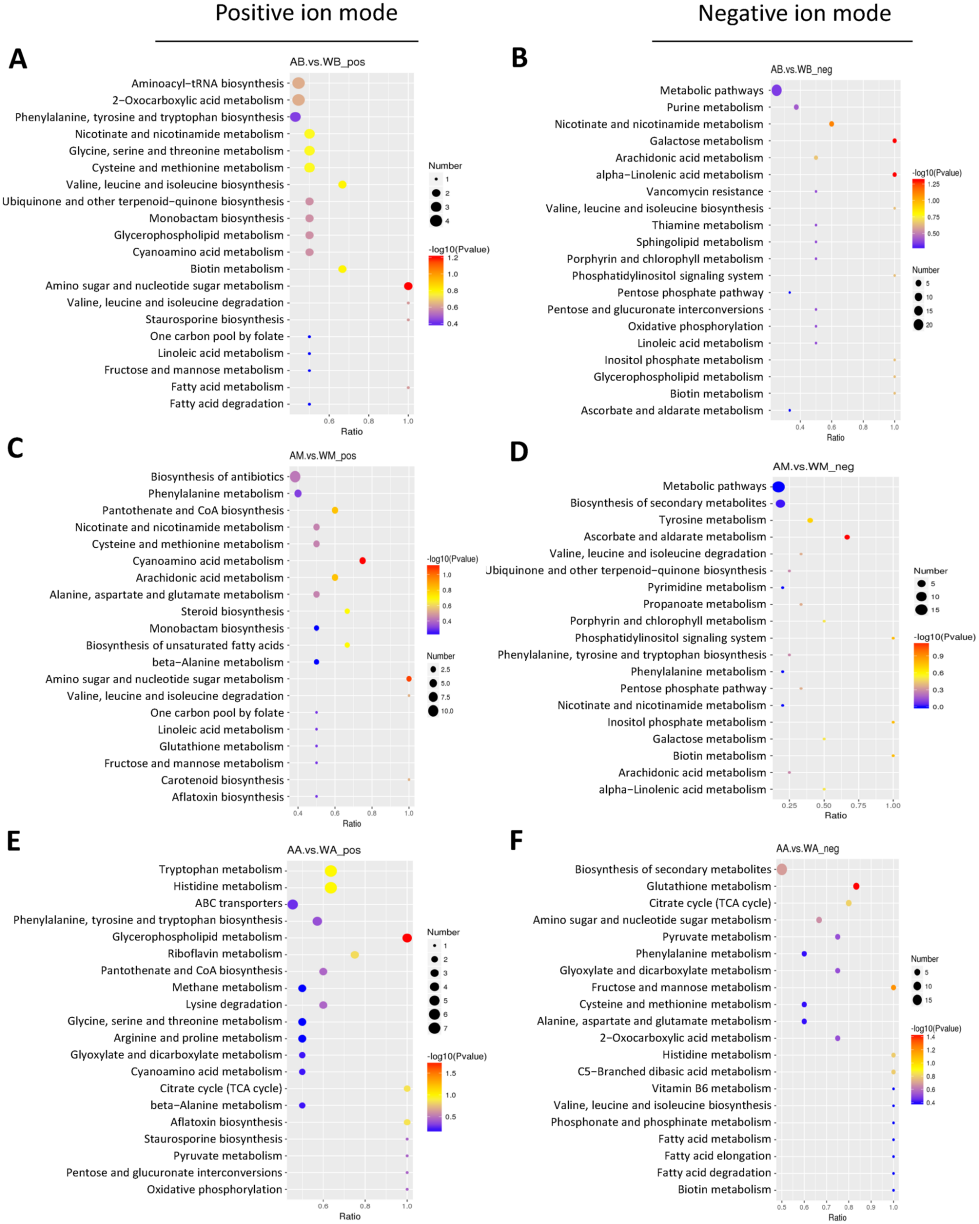
KEGG enrichment bubble diagram of metabolites of cultivated and wild *Inonotus hispidus* in different growth periods. (**A**) AB vs. WB under the positive ion mode, (**B**) AB vs. WB under the negative ion mode, (**C**) AM vs. WM under the positive ion mode, (**D**) AM vs. WM under the negative ion mode, (**E**) AA vs. WA under the positive ion mode, (**F**) AA vs. WA under the negative ion mode. Note: AB, AM, and AA are samples of fruiting bodies of cultivated *Inonotus hispidus* in budding, mature and aging growth periods, respectively. WB, WM, and WA are samples of fruiting bodies of wild *Inonotus hispidus* in budding, mature and aging growth periods, respectively.

In order to further understand the differences in metabolic networks among samples, the annotated differential metabolites were submitted to the KEGG website for metabolic pathway enrichment analysis. The enrichment pathway of differential metabolites between cultivated and wild *I. hispidus* in the bud stage, mature stage, and aging stage (Fig. 5). It mainly includes eight main metabolic pathways, such as steroid biosynthesis, biosynthesis of amino acids, organic acid metabolism, glycine, serine and threonine metabolism, tricarboxylic acid cycle, glycolysis, purine metabolism, and pyrimidine metabolism, as well as some secondary metabolic pathways. Although artificial cultivation and wild *I. hispidus* have the same kinds of metabolites, the enrichment degree of metabolites is different in different physiological maturity stages.

### Ergosterol peroxide and (22E)-ergosta-4,6,8(14),22-tetraen-3-one content in *Inonotus hispidus* samples collected at different physiological maturity stages

**Figure 6:**
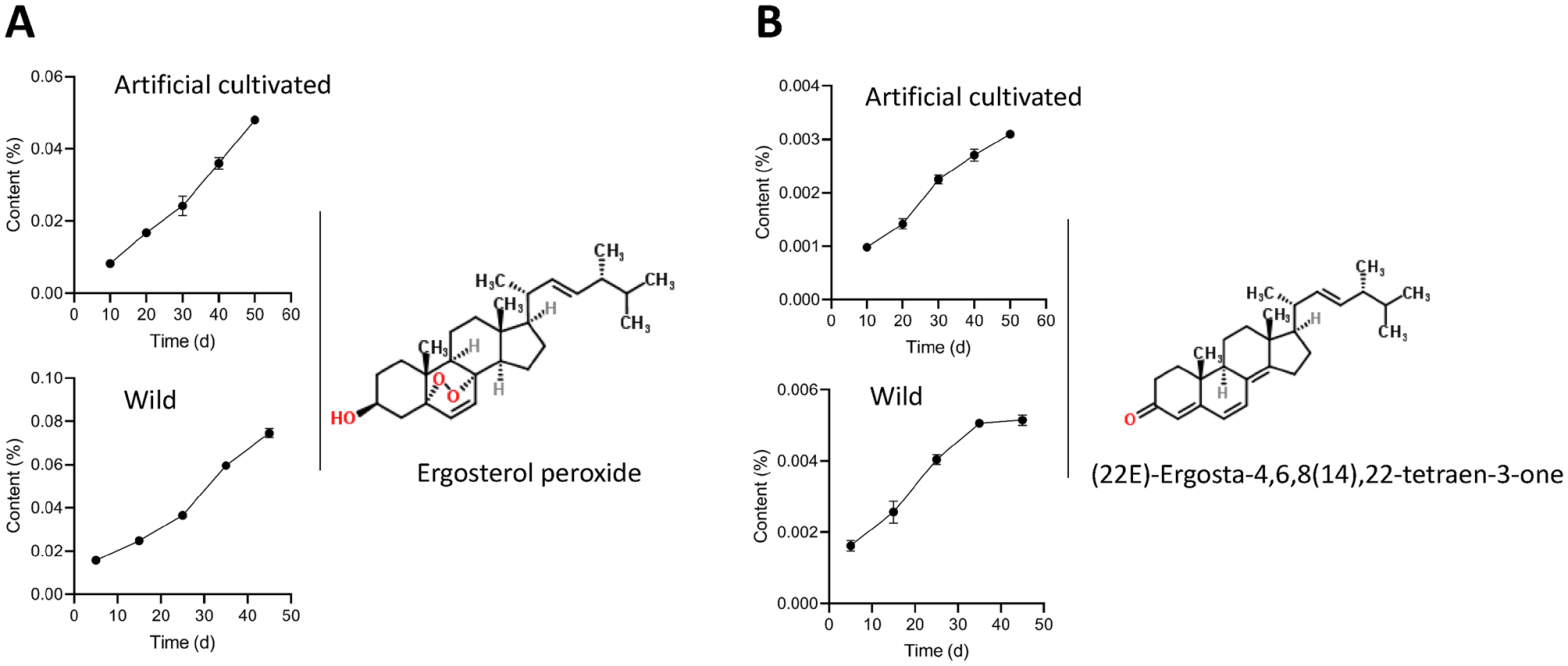
Contents of ergosterol peroxide and (22E)-ergosta-4,6,8(14),22-tetraen-3-one in fruiting bodies of *Inonotus hispidus* in different growth periods. (**A**) Ergosterol peroxide, (**B**) (22E)-ergosta-4,6,8(14),22-tetraen-3-one.

Among the annotated metabolites, ergosterol peroxide attracted our attention because of the high fold-change difference in its abundance in different grow period samples. The abundance of ergosterol peroxide increased significantly, which prompted us to consider whether this metabolite can be used as an indicator of the physiological maturity of fruiting bodies. Therefore, the fruiting body samples were collected on days 10, 20, 30, 40, and 50, and the contents of ergosterol peroxide were determined by High Performance Liquid Chromatography (HPLC) according to the method reported in the literature. During the period of day 1 to day 20, the fruiting body of *I. hispidus* was golden in the budding period, mature on days 20 to day 50, yellowish-brown in the fruiting body, and gray-black in the aging period after day 50. During the whole process of growth and development, the metabolites with continuous decrease or increase in the abundance of metabolites may become an indicator of fruiting body maturity. It was found that the content of ergosterol peroxide was low in the budding period and could hardly be detected, but it was higher in the aged fruiting body. It is speculated that ergosterol peroxide can be used as a potential indicator to identify whether the fruiting body has reached physiological maturity. The content of ergosterol peroxide was 0.008% on days 10 of the fruiting body of cultivated *I. hispidus*, 0.024% on day 30 of maturity, 0.048% on day 50 of aging, and with the extension of the fruiting body growth period, the content of ergosterol peroxide increased and showed an upward trend (Fig. 6A).

In addition, the results of HPLC showed that the content of (22E)-ergosta-4,6,8(14),22-tetraen-3-one gradually increased with the physiological maturity of *I. hispidus* mushroom. The compound (22E)-ergosta-4,6,8(14),22-tetraen-3-one has strong fluorescence under 365 nm ultraviolet lamp, so it was easy to be identified and determined. The content was 0.001% at the budding period on day 10 of the cultivated fruiting body, 0.002% on day 30 of maturity, and only 0.003% on day 50 aging period. In this study, the contents of ergosterol peroxide, and (22E)-ergosta-4,6,8(14),22-tetraen-3-one in the fruiting bodies of wild and cultivated *I. hispidus* showed some regular and linear changes in the process of physiological maturation (Fig. 6B).

## Discussion

This work investigated the fruiting bodies' metabolites change regulation and potential maturating indicators to distinguish different quality *I. hispidus*. The results showed that there were significant differences in the composition of metabolites in the fruiting bodies of *I. hispidus* in different growth periods, which would lead to different medicinal values. Therefore, different growth periods have a significant impact on the quality of it, and it is necessary to further study and clarify the differences. In our analysis, a total of 1358 metabolites were annotated from *I. hispidus* fruiting bodies. Based on the metabolic profile analysis of the KEGG database (https://www.genome.jp/kegg/pathway.html) and LIPIDMaps database (http://www.lipidmaps.org/), including 20 sugars, 12 amino acids, 24 oligopeptides, 23 steroids, 31 phenols, 12 flavones, 4 isoflavones, and 10 flavonoids and 11 terpenes were annotated. There are significant differences in metabolites in the process of physiological maturation of mushroom *I. hispidus*, which is mainly reflected in the change of abundance rather than in kinds.

Among the metabolites of *I. hispidus*, sugars, amino acids, oligopeptides, steroids, polyphenols, flavonoids, and terpenoids have been reported to have pharmacology activity. Therefore, these metabolites are mainly listed in this paper. Sugars are one of the effective components in *I. hispidus*, modern pharmacological studies have shown that *I. hispidus* sugars have protective effects on acute alcoholic liver injury in mice^1^. Amino acids as one of the important indicators of food nutritional value evaluation^28,29^, and have many pharmacological activities, such as L-leucine is used in the clinical treatment of liver disease^30^. Modern pharmacological studies show that serine plays a role in relieving chronic low-back and knee pain in adults^31^, leucine can by promoting insulin and Glucagon-Like Peptide 1 (GLP-1) secretion. With physiological maturity, the relative abundance of amino acids such as lysine, threonine, asparagine, serine and glutamic acid and histidine in the fruiting body decreased gradually. The oligopeptide is a biochemical substance between amino acid and protein, which is composed of 2 to 15 amino acids. In this study, it is found that the content of oligopeptides is abundant, especially with the physiological maturity of the fruiting body, the abundance of Glu-Val-Phe increases significantly. On the contrary, the abundance of Tyr-Tyr decreases significantly with the physiological maturity of the fruiting body. Steroids and phenols in *I. hispidus* are important active components, which have antioxidants^2^, anti-inflammatory^6^, and bacteriostatic activities^6^. In recent years, flavonoids and terpenoids as two important kinds of metabolites in *I. hispidus* have been widely concerned and studied^2^. Modern pharmacological studies have shown that flavonoids have antioxidant potential and antimicrobial activity^32^. Some terpenoids have the effects of antioxidation, liver protection, immunity enhancement, anti-tumor, and so on. Such as betulin, which has anti-inflammatory^33^, antiviral^34^, and other activities^35^. With physiological maturity, the relative abundance of betulin in the fruiting body decreased gradually. These findings suggest that the abundance of metabolites showed some regular changes with the fruiting bodies maturating of *I. hispidus*. When using these fruiting bodies as raw materials for functional foods and drugs, special attention should be paid to the changes of the abundance of metabolites in the fruiting bodies of *I. hispidus*.

From the perspective of primary metabolites and secondary metabolites, the changes of eight main metabolites of wild and cultivated *I. hispidus* mushroom during different growth stages were compared and analyzed. The result showed that the abundance of total polysaccharides, crude fat, total flavonoids, and total terpenes increased at first and then decreased. Interestingly, total amino acids, crude protein, and total polyphenols decreased during the growth periods, while the total steroids increased linearly. In this study, it is worth noting that the content of 8 kinds of primary and secondary metabolites of cultivated *I. hispidus* mushroom is significantly higher than that of wild, which may be related to the fact that water, temperature, nutrients, and other factors in the artificial cultivation environment are more suitable for the growth of it. Compared with the growth environment conditions of wild *I. hispidus*, cultivated has more stable growth environment conditions, and is more stable and unified in soil nutritional status, temperature, humidity, precipitation, and so on. These may be some important reasons for the high quality of cultivated. Metabolite profiles are highly affected by environmental conditions, but it needs to be explained by further research. On the one hand, the changes of metabolites in cultivated were more stable and the content of metabolites higher than the wild ones, which is more suitable for the function of food and medicine. On the other hand, cultivated *I. hispidus* is easier to control the growth environment conditions, standardized production, and easy to obtain high yield. These results suggested that cultivated *I. hispidus* may be more suitable for the needs of industrial products. From the perspective of primary metabolites and secondary metabolites, the relative contents of total amino acids and total polysaccharides decrease gradually with the physiological maturity of fruiting bodies, if the sugars or amino acids in *I. hispidus* are used in industry, then the maximum benefit may be obtained in the bud stage, of course, the yield needs to be considered. If steroids are used in industry, and the content of steroids increases with the physiological maturity of the *I. hispidus* fruiting bodies, then the aging *I. hispidus* fruiting bodies may be more suitable. This study showed that there were significant differences in the relative abundance of primary and secondary metabolites in the fruiting bodies of *I. hispidus* in different growth periods. This study provides a scientific basis for selecting the best *I. hispidus* fruiting bodies in the growing period according to the demand.

In addition, a lack of measurable indicators that can characterize the physiological maturity of *I. hispidus*, leads to the subjective evaluation of the physiological maturity and the fluctuation of the yield and quality of *I. hispidus* and the difference of production efficiency of different producers. Therefore, there is an urgent need for an indicator of fruiting body maturation, to provide a basis for production and research. In our study, the content of ergosterol peroxide and (22E)-ergosta-4,6,8(14),22-tetraen-3-one in fruiting bodies showed a regular change during physiological maturation. Almost no ergosterol peroxide and (22E)-ergosta-4,6,8(14),22-tetraen-3-one were detected in fruiting bodies collected on day 0 of the wild and cultivated *I. hispidus*, but its content was extremely high in physiologically mature fruiting bodies. We deduced that ergosterol peroxide and (22E)-ergosta-4,6,8(14),22-tetraen-3-one may be used as potential indicators to identify whether fruiting bodies have reached physiological maturity. However, the utility of ergosterol peroxide and (22E)-ergosta-4,6,8(14),22-tetraen-3-one content as an indicator of the physiological maturation of mycelia was not determined, and this question merits additional fruiting experiments.

In summary, this study showed that with the fruiting bodies maturating, the abundance of metabolites showed some regular changes. The cultivated and wild *I. hispidus* mushroom has the same 1358 metabolites, but the metabolites showed distinct changes in abundance in cultivated and wild. It is worth noting that mass spectrometry can’t detect all the metabolites, not because the mass spectrometry is not sensitive enough, but because mass spectrometry can only detect ionized substances, but some metabolites can’t be ionized in the mass spectrometer. Nuclear magnetic resonance (NMR) is needed to make up for the lack of chromatography in the future, and further research is needed^36–38^. Through the metabonomic and natural product research combined, the content of ergosterol peroxide and (22E)-ergosta-4,6,8(14),22-tetraen-3-one showed a linear trend increase, which indicated the feasibility of their use as an indicator for fruiting body maturation, but whether this indicator can be applied for the commercial production of mushroom *I. hispidus* requires the verification of large-scale fruiting experiences. The metabolites of *I. hispidus* fruiting bodies were different in different growth stages, so the quality was different. However, the maturity of the fruiting body can be determined by peroxidation of ergosterol and (22E)-ergosta-4,6,8(14),22-tetraen-3-one, and the quality of the fruiting body can be screened indirectly. Our research provided an atheoretical basis for quality evaluation and comprehensive utilization of the mushroom *I. hispidus* from cultivated and wild.

## Materials and methods

### Chemicals

All chemicals used in this study were of chromatographic grade for liquid chromatography. Acetonitrile, methanol, and formic acid were purchased from Merck (Darmstadt, Germany). Acetic acid and methyl alcohol were from Tedia (Tedia Co., Ohio, USA). Deionized water was purified by a Milli-Q water purification system (Millipore, Billerica, MA, USA).

### Samples

Wild *I. hispidus* mushrooms were collected from the ancient mulberry protection forest in Xiajin County, Dezhou City, Shandong Province, China (36°59′ N, 115°11′ E). Samples were randomly collected and immediately frozen in liquid nitrogen. The cultivated *I. hispidus* mushroom was collected from Xiajin Sanghuang Biotechnology Co., Ltd. 6 replicate samples (sampling position: middle part) were collected at each time point and frozen in liquid nitrogen immediately. All samples are stored at − 80 °C until metabolomics analysis. The specimens (No. HMJAU58767) are deposited in the Key Laboratory of Medicinal Fungal Resources and Development and Utilization, Jilin agricultural university.

### Metabolites extraction

Crushed tissues (100 mg) were placed in the Eppendorf tube (EP tube) and resuspended with prechilled 80% methanol and 0.1% formic acid. After the vortex shock, the samples were incubated on ice for 5 min and then were centrifuged at 15,000×*g*, 4 °C for 20 min. To reduce the matrix effect, better detect metabolites and optimize the peak shape, 300 μL supernatant and 150 μL LC–MS grade water. were diluted to 53% methanol content. The samples were subsequently transferred to a fresh Eppendorf tube and then were centrifuged at 15,000×*g*, 4 °C for 20 min. Finally, the supernatant was injected into the UHPLC-MS/MS system analysis^39^.

### UHPLC-MS/MS analysis

UHPLC-MS/MS analysis refers to previously methods^40^. UHPLC-MS/MS analyses were performed using a Vanquish UHPLC system (Thermo Fisher, Germany) coupled with an Orbitrap Q Exactive HF mass spectrometer (Thermo Fisher, Germany). Samples were injected onto a Hypesil Gold column (100 × 2.1 mm, 1.9 μm) using a 17 min linear gradient at a flow rate of 0.2 mL/min. The eluents for the positive polarity mode were eluent A (0.1% FA in Water) and eluent B (Methanol). The eluents for the negative polarity mode were eluent A (5 mM ammonium acetate, pH 9.0) and eluent B (Methanol). The solvent gradient was set as follows: 2% B, 1.5 min; 2–100% B, 12.0 min; 100% B, 14.0 min; 100–2% B, 14.1 min; 2% B, 17 min. Q Exactive HF mass spectrometer was operated in positive/negative polarity mode with a spray voltage of 3.2 kV, capillary temperature of 320 °C, sheath gas flow rate of 40 arb, and aux gas flow rate of 10 arb.

### Total polysaccharides, total amino acids, crude proteins, total sterols, total polyphenols, total flavonoids, terpenes, and crude fat were determined in fruiting bodies of Inonotus hispidus during different growth periods

Through the analysis of the metabolic profiling of the fruiting bodies of wild and cultivated *I. hispidus* in different growth periods, the complex chemical composition and its change trend line were shown, but the practical application may involve a macroscopic overall quantitative analysis. Therefore, the contents of total polysaccharides, total amino acids, crude protein, total steroids, polyphenols, total flavonoids, terpenoids, and crude fat in wild and cultivated *I. hispidus* fruiting bodies were analyzed in this study.

### Data processing and metabolite annotation

Data processing and metabolite annotation refer to previously methods^23^. The raw data files generated by UHPLC-MS/MS were processed using the Compound Discoverer 3.1 (CD3.1, Thermo Fisher, USA) to perform peak alignment, peak picking, and quantitation for each metabolite. The main parameters were set as follows: retention time tolerance, 0.2 min; actual mass tolerance, 5 ppm; signal intensity tolerance, 30%; signal/noise ratio, 3; and minimum intensity, 100,000. After that, peak intensities were normalized to the total spectral intensity. According to the formal definition of metabolites annotation and annotation developed by the Chemical Analysis Working Group of the Metabolomics Standards Initiative (MSI) working Group on Chemical Analysis^41^, this study annotates metabolites detected by non-targeted metabonomic scans. The normalized data were used to predict the molecular formula based on additive ions, molecular ion peaks, and fragment ions. And then peaks were matched with the mzCloud (https://www.mzcloud.org/), mzVault, and MassList database to obtain the accurate qualitative and relative quantitative results. Statistical analyses were performed using the statistical software R (R version R-3.4.3), Python (Python 2.7.6 version), and CentOS (CentOS release 6.6), When data were not normally distributed, normal transformations were attempted using of area normalization method.

### Metabonomic data analysis

Data were normalized for internal consistency by processing the constant weight of the solvent per volume of each sample. The internal consistency of the data is normalized data were scaled to the median value for each compound, then missing values were imputed with the minimum detected value for that compound. Moreover, a variety of curation procedures were carried out to ensure that a high-quality data set was made available for statistical analysis and data interpretation. The QC and curation processes were designed to ensure accurate and consistent annotation of true chemical entities and to remove those representing system artifacts, misassignments, and background noise. Library matches for each compound were checked for each sample and corrected if necessary. Metabonomic data analysis refers to previously methods^24,26,42–44^. These metabolites were annotated using the Kyoto Encyclopedia of Genes and Genomes (KEGG) database (https://www.genome.jp/kegg/pathway.html), Human Metabolome Database (HMDB) (https://hmdb.ca/metabolites), and LIPIDMaps database (http://www.lipidmaps.org/).

The data analysis of PCA and PLS-DA were performed using software python (Python-3.5.0), and the charts were drawn by software R (R-3.4.3). We applied univariate analysis (t-test) to calculate the statistical significance (P-value). The metabolites with VIP > 1 and P-value < 0.05 and fold change ≥ 2 or FC ≤ 0.5 were considered to be differential metabolites. Volcano plots were used to filter metabolites of interest-based on log2(FoldChange) and -log10(p-value) of metabolites.

For clustering heat maps, the data were normalized using z-scores of the intensity areas of differential metabolites and were plotted by the Pheatmap package in R language. A statistically significant correlation between differential metabolites was calculated by R language. P-value < 0.05 was considered as statistically significant and correlation plots were plotted by corrplot package in R language. The metabolic pathways enrichment of differential metabolites was performed, when the ratio was satisfied by x/n > y/N, metabolic pathways were considered as enrichment, when the P-value of metabolic pathway < 0.05, metabolic pathways were considered as statistically significant enrichment. For line charts and tables, the data were normalized using Microsoft Office Excel 2019 software, and the chemical structure formula was made by ChemDraw 15 software.

## Supplementary Information

Supplementary Information 1.

Supplementary Information 2.

Supplementary Information 3.

Supplementary Information 4.
