# Supplementary material for: The regular pattern of metabolite changes in mushroom Inonotus hispidus in different growth periods and exploration of their indicator compounds
Source: Sci Rep. 2022 Aug 23;12:14354. doi: 10.1038/s41598-022-18631-9 (PMC9399111; doi:10.1038/s41598-022-18631-9)
Supplement: Supplementary file 4 — Supplementary Information 4. [file 41598_2022_18631_MOESM4_ESM.docx]

Graphic for manuscript
